# Supplementary material for: The promise and challenge of spatial inference with the full ancestral recombination graph under Brownian motion
Source: G3 (Bethesda). 2025 Sep 22;15(11):jkaf214. doi: 10.1093/g3journal/jkaf214 (PMC12608074; doi:10.1093/g3journal/jkaf214)
Supplement: jkaf214_Supplementary_Data [file jkaf214_supplementary_data.pdf]

Supplementary material for "The promise and challenge of spatial inference with the full ancestral recombination graph under Brownian motion"

Puneeth Deraje[1†*], James Kitchens[2†*], Graham Coop[2‡], and Matthew M. Osmond[1‡]

[†]These authors contributed equally to this work
[‡]These authors equally supervised this work
[1]Department of Ecology & Evolutionary Biology, University of Toronto
[2]Department of Evolution & Ecology and Center for Population Biology, University of California - Davis
[*]Corresponding authors: puneeth.deraje@mail.utoronto.ca and jkitchens@ucdavis.edu

S1 Likelihood of sample locations

We start by assuming that the random displacements along each edge of the ARG, B_{edge}, are independent. Hence, each path, from a root to a sample, gives a unique distribution for the location of a sample, even if two or more paths end at the same sample. Given we have n_p unique paths, we therefore get the distributions for n_p sample locations, $\overrightarrow{L_p}$. In order to get the distribution of the actual n_s sample locations, $\overrightarrow{L}$, we condition on the loop conditions, η_{loops},

$$f_{\overrightarrow{L_p}|\eta_{\text{loops}}}(\vec{\ell_p}) = \frac{f_{\overrightarrow{L_p}}(\vec{\ell_p} \cap \eta_{\text{loops}})}{f_{\overrightarrow{L_p}}(\eta_{\text{loops}})}, \tag{S1}$$

where we use the shorthand $f_{\overrightarrow{L_p}}(\eta_{\text{loops}}) = \int_{\eta_{\text{loops}}} f_{\overrightarrow{L_p}}(\ell)\mathrm{d}\ell$ for the probability density of the loop conditions.

In Section S2 we show that the loop and path conditions are the same, $\eta_{\text{loops}} = \eta_{\text{paths}}$. Since η_{paths} conditions the paths that end at the same sample to have identical locations, the numerator above becomes $f_{\overrightarrow{L_p}}(\vec{\ell}_p \cap \eta_{\text{loops}}) = f_{\overrightarrow{L_p}}(\vec{\ell}_p \cap \eta_{\text{paths}}) = f_{\overrightarrow{L_p}}(\mathbf{P}\vec{\ell}\,)$, where $\mathbf{P}$ is the path-sample matrix (a $n_p \times n_s$ matrix whose ij^{th} entry is 1 if the ith path ends at sample j). Then the distribution of path locations becomes

$$f_{\overrightarrow{L_p}|\eta_{\text{loops}}}(\vec{\ell}_p) = \mathbb{1}[\vec{\ell}_p = \mathbf{P}\vec{\ell}\,] \frac{f_{\overrightarrow{L_p}}(\mathbf{P}\vec{\ell}\,)}{f_{\overrightarrow{L_p}}(\eta_{\text{paths}})}, \tag{S2}$$

where $\mathbb{1}[\vec{\ell}_p = \mathbf{P}\vec{\ell}\,]$ is an indicator function that is 1 if $\vec{\ell}_p = \mathbf{P}\vec{\ell}$ and 0 otherwise. This ensures that the probability density is 0 whenever any two paths ending at the same sample have different locations. We will now compute this distribution for the case of a single root and then do the more general multiple-root case.

S1.1 Single root

When the ARG has a single root, located at μ, the locations of path ends, $\overrightarrow{L_p}$, is multivariate normal with mean $\mu\mathbb{1}_{n_p}$ and covariance matrix $\sigma^2\mathbf{S}_p$, where σ^2 is the dispersal rate and $\mathbf{S}_p$ the path matrix (shared time between each pair of paths). Therefore the numerator of Equation S2 is

$$f_{\overrightarrow{L_p}}(\mathbf{P}\vec{\ell}\,) = \int \frac{1}{\sqrt{(2\pi\sigma^2)^{\text{rnk}(\mathbf{S}_p)}|\mathbf{S}_p|}} \exp\left(-\frac{(\vec{\ell} - \mu\mathbb{1}_{n_s})^T\mathbf{P}^T\mathbf{S}_p^{g-}\mathbf{P}(\vec{\ell} - \mu\mathbb{1}_{n_s})}{2\sigma^2}\right) d\vec{\ell}, \tag{S3}$$

where $\mathbf{S}_p^{g-}$ is the generalized inverse of $\mathbf{S}_p$ and $\text{rnk}(\mathbf{S}_p)$ is the rank of $\mathbf{S}_p$ (which may be less than n_p). Meanwhile the denominator is

$$\begin{aligned} f_{\overrightarrow{L_p}}(\eta_{\text{paths}}) &= \int f_{\overrightarrow{L_p}}(\vec{\ell}_p \cap \eta_{\text{paths}}) d\vec{\ell}_p && \text{(S4)} \\ &= \int f_{\overrightarrow{L_p}}(\mathbf{P}\vec{\ell}\,) d\vec{\ell} && \text{(S5)} \\ &= \int \frac{1}{\sqrt{(2\pi\sigma^2)^{\text{rnk}\,\mathbf{S}_p}|\mathbf{S}_p|}} \exp\left(-\frac{(\vec{\ell} - \mu\mathbb{1}_{n_s})^T\mathbf{P}^T\mathbf{S}_p^{g-}\mathbf{P}(\vec{\ell} - \mu\mathbb{1}_{n_s})}{2\sigma^2}\right) d\vec{\ell} && \text{(S6)} \\ &= \frac{\sqrt{(2\pi\sigma^2)^{n_s}|\mathbf{S}|}}{\sqrt{(2\pi\sigma^2)^{\text{rnk}\,\mathbf{S}_p}|\mathbf{S}_p|}}, && \text{(S7)} \end{aligned}$$

where $\mathbf{S} = (\mathbf{P}^T\mathbf{S}_p^{g-}\mathbf{P})^{-1}$ is the sample covariance matrix.

The probability density of the path locations, conditional on the loops (equivalently, paths) meeting, is then

$$f_{\overrightarrow{L_p}|\eta_{\text{loops}}}(\vec{\ell_p}) = \mathbb{1}[\vec{\ell_p} = \mathbf{P}\vec{\ell}\,]\frac{1}{\sqrt{(2\pi\sigma^2)^{n_s}|\mathbf{S}|}}\exp\left(-\frac{(\vec{\ell} - \mu\mathbb{1}_{n_s})^T\mathbf{S}^{-1}(\vec{\ell} - \mu\mathbb{1}_{n_s})}{2\sigma^2}\right), \tag{S8}$$

which is the probability density of a multivariate normal random variable with mean $\mu\mathbb{1}_{n_s}$ and covariance matrix $\sigma^2\mathbf{S}$. This is the likelihood of sample locations, $\vec{L}$, given Brownian motion down the ARG. The maximum likelihood estimates of dispersal rate and root location are then given by

$$\widehat{\mu} = (\mathbb{1}_{n_p}\mathbf{S}_p^{g-}\mathbb{1}_{n_p})^{-1}\mathbb{1}_{n_p}\mathbf{S}_p^{g-}\vec{\ell^*} \tag{S9}$$

$$\widehat{\sigma^2} = \frac{(\vec{\ell^*} - \mu\mathbb{1}_{n_s})^T\mathbf{P}^T\mathbf{S}_p^{g-}\mathbf{P}(\vec{\ell^*} - \mu\mathbb{1}_{n_s})}{n_s}, \tag{S10}$$

where $\vec{\ell^*}$ are the observed sample locations.

S1.2 Multiple Roots

We next want to generalize this to multiple roots, which occurs when we chop off an ARG more recently than the grand most recent common ancestor (Figure S1). Let n_r be the number of roots, $\vec{\mu}$ be $n_r \times 1$ vector of root locations, and $\mathbf{R}$ be the $n_p \times n_r$ path-root matrix (the i, j^{th} entry is 1 if path i starts at root j, otherwise 0). Then the (unconditioned) probability distribution of the path locations, $\overrightarrow{L_p}$, is multivariate normal with mean $\mathbf{R}\vec{\mu}$ and covariance $\sigma^2\mathbf{S}_p$. Now, as in the single root case, we want to find the distribution of the path locations conditioned on the paths meeting at the samples, η_{paths}.

As with a single root, the numerator of equation S2 can be written $f_{\overrightarrow{L_p}}(\vec{\ell_p} \cap \eta_{\text{loops}}) = f_{\overrightarrow{L_p}}(\vec{\ell_p} \cap \eta_{\text{paths}}) = f_{\overrightarrow{L_p}}(\mathbf{P}\vec{\ell}\,)$, but now this is

$$f_{\overrightarrow{L_p}}(\mathbf{P}\vec{\ell}\,) = \frac{\exp[-\frac{1}{2\sigma^2}(\mathbf{P}\vec{\ell} - \mathbf{R}\vec{\mu})^{\mathrm{T}}\mathbf{S}_p^{g-}(\mathbf{P}\vec{\ell} - \mathbf{R}\vec{\mu})]}{\sqrt{(2\pi\sigma^2)^{n_p}|\mathbf{S}_p|}}. \tag{S11}$$

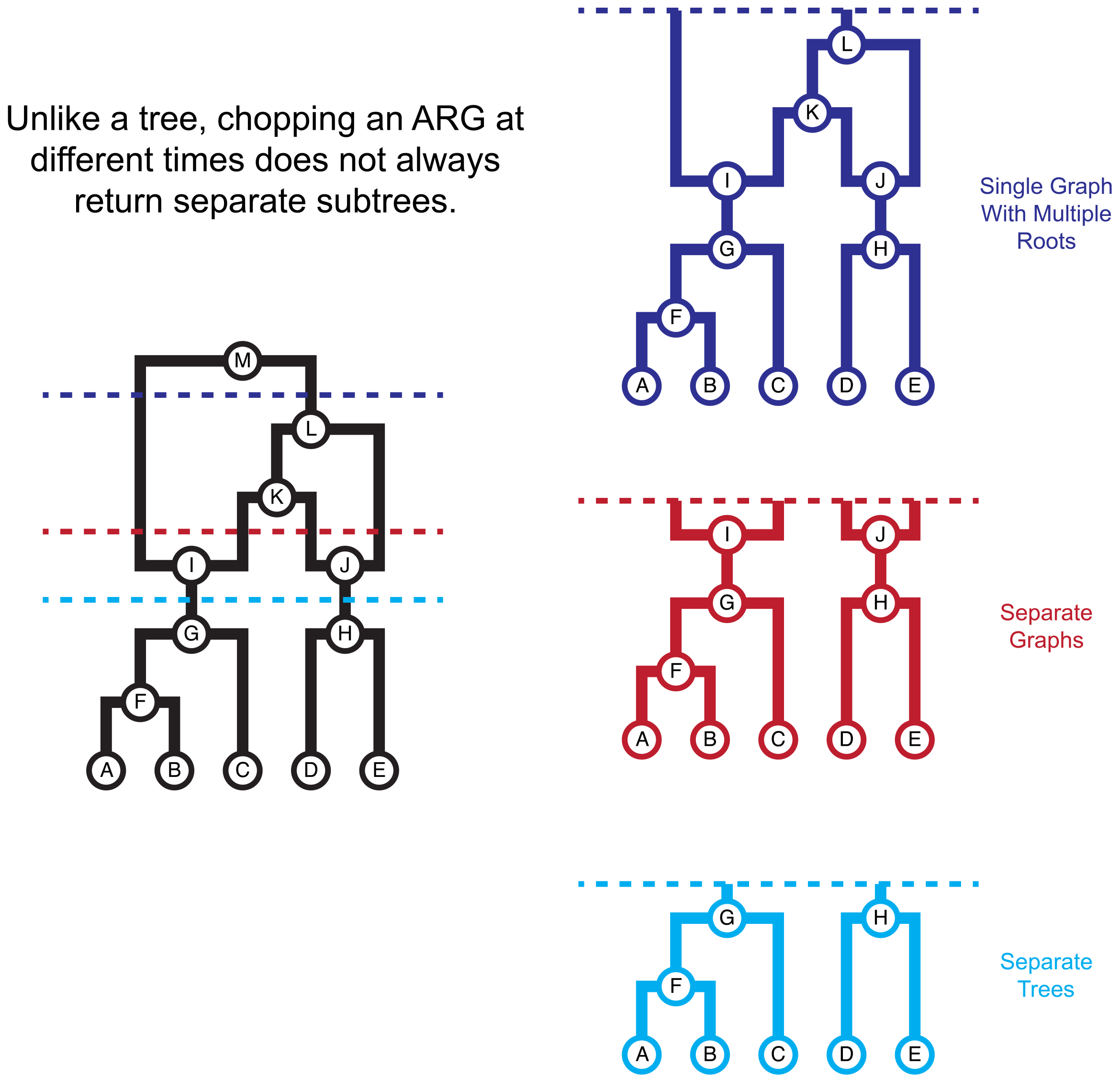

Figure S1: **Multiple roots**. Cartoon for the various scenarios that may occur when chopping an ARG below its grand most recent common ancestor.

Similarly, the denominator becomes

$$
\begin{aligned}
f_{\overrightarrow{L_p}}(\eta_{\text{paths}}) &= \int f_{\overrightarrow{L_p}}(\mathbf{P}\vec{\ell}\,)d\vec{\ell} && \text{(S12)}\\
&= \int \frac{\exp[-\frac{1}{2\sigma^2}(\mathbf{P}\vec{\ell}\, - \mathbf{R}\vec{\mu})^{\mathrm{T}}\mathbf{S}_p^{g-}(\mathbf{P}\vec{\ell}\, - \mathbf{R}\vec{\mu})]}{\sqrt{(2\pi\sigma^2)^{n_p}|\mathbf{S}_p|}}d\vec{\ell}\,. && \text{(S13)}
\end{aligned}
$$

To find this integral we multiply and divide by a constant to make the integrand a probability density for $\vec{\ell}$. In order to do that, note that the term in the exponent can be expanded like

$$
\begin{aligned}
& (\mathbf{P}\vec{\ell}\, - \mathbf{R}\vec{\mu})^{\mathrm{T}}\mathbf{S}_p^{g-}(\mathbf{P}\vec{\ell}\, - \mathbf{R}\vec{\mu}) && \text{(S14)}\\
= & \; \vec{\ell}\,^{\mathrm{T}}\mathbf{P}^{\mathrm{T}}\mathbf{S}_p^{g-}\mathbf{P}\vec{\ell}\, - 2\vec{\mu}^{\mathrm{T}}\mathbf{R}^{\mathrm{T}}\mathbf{S}_p^{g-}\mathbf{P}\vec{\ell}\, + \vec{\mu}^{\mathrm{T}}\mathbf{R}^{\mathrm{T}}\mathbf{S}_p^{g-}\mathbf{R}\vec{\mu} && \text{(S15)}\\
= & \; \vec{\ell}\,^{\mathrm{T}}[\mathbf{P},\mathbf{P}]\vec{\ell}\, - 2\vec{\mu}^{\mathrm{T}}[\mathbf{R},\mathbf{P}]\vec{\ell}\, + \vec{\mu}^{\mathrm{T}}[\mathbf{R},\mathbf{R}]\vec{\mu} && \text{(S16)}\\
= & \; \vec{\ell}\,^{\mathrm{T}}[\mathbf{P},\mathbf{P}]\vec{\ell}\, - 2\vec{\mu}^{\mathrm{T}}[\mathbf{R},\mathbf{P}][\mathbf{P},\mathbf{P}]^{-1}[\mathbf{P},\mathbf{P}]\vec{\ell}\, + \vec{\mu}^{\mathrm{T}}[\mathbf{R},\mathbf{R}]\vec{\mu} && \text{(S17)}\\
= & \; \vec{\ell}\,^{\mathrm{T}}[\mathbf{P},\mathbf{P}]\vec{\ell}\, - 2\vec{\mu}_\ell^{\mathrm{T}}[\mathbf{P},\mathbf{P}]\vec{\ell}\, + \vec{\mu}^{\mathrm{T}}[\mathbf{R},\mathbf{R}]\vec{\mu} && \text{(S18)}\\
= & \; \vec{\ell}\,^{\mathrm{T}}[\mathbf{P},\mathbf{P}]\vec{\ell}\, - 2\vec{\mu}_\ell^{\mathrm{T}}[\mathbf{P},\mathbf{P}]\vec{\ell}\, + \vec{\mu}_\ell^{\mathrm{T}}[\mathbf{P},\mathbf{P}]\vec{\mu}_\ell - \vec{\mu}_\ell^{\mathrm{T}}[\mathbf{P},\mathbf{P}]\vec{\mu}_\ell + ... && \text{(S19)}\\
& ...\; \vec{\mu}^{\mathrm{T}}[\mathbf{R},\mathbf{R}]\vec{\mu} && \text{(S20)}\\
= & \; (\vec{\ell}\, - \vec{\mu}_\ell)^{\mathrm{T}}[\mathbf{P},\mathbf{P}](\vec{\ell}\, - \vec{\mu}_\ell) - \vec{\mu}_\ell^{\mathrm{T}}[\mathbf{P},\mathbf{P}]\vec{\mu}_\ell + \vec{\mu}^{\mathrm{T}}[\mathbf{R},\mathbf{R}]\vec{\mu} && \text{(S21)}\\
= & \; (\vec{\ell}\, - \vec{\mu}_\ell)^{\mathrm{T}}[\mathbf{P},\mathbf{P}](\vec{\ell}\, - \vec{\mu}_\ell) + ... && \text{(S22)}\\
& ...\; \vec{\mu}^{\mathrm{T}}\big([\mathbf{R},\mathbf{R}] - [\mathbf{R},\mathbf{P}][\mathbf{P},\mathbf{P}]^{-1}[\mathbf{P},\mathbf{R}]\big)\vec{\mu}. && \text{(S23)}
\end{aligned}
$$

In step 1 above we have used $\vec{\ell}\,^{\mathrm{T}}\mathbf{P}^{\mathrm{T}}\mathbf{S}_p^{g-}\mathbf{R}\vec{\mu} = \vec{\mu}^{\mathrm{T}}\mathbf{R}^{\mathrm{T}}\mathbf{S}_p^{g-}\mathbf{P}\vec{\ell}$, as these are 1×1 matrices and therefore are the transpose of each other. We have also used the shorthand $[\mathbf{A},\mathbf{B}] = \mathbf{A}^{\mathrm{T}}\mathbf{S}_p^{g-}\mathbf{B}$ and introduced $\vec{\mu}_\ell = [\mathbf{P},\mathbf{P}]^{-1}[\mathbf{P},\mathbf{R}]\vec{\mu}$, a $n \times 1$ vector which will correspond to the expectation of the sample locations (as shown below). Using this expansion, we first define

$$
\begin{aligned}
N_{\text{old}} &= \frac{\exp\Big[-\dfrac{1}{2\sigma^2}\vec{\mu}^{\mathrm{T}}\big([\mathbf{R},\mathbf{R}] - [\mathbf{R},\mathbf{P}][\mathbf{P},\mathbf{P}]^{-1}[\mathbf{P},\mathbf{R}]\big)\vec{\mu}\Big]}{\sqrt{(2\pi\sigma^2)^{n_p}|\mathbf{S}_p|}} && \text{(S24)}\\
N_{\text{new}} &= \sqrt{(2\pi\sigma^2)^{n_s}|[\mathbf{P},\mathbf{P}]^{-1}|} && \text{(S25)}
\end{aligned}
$$

and write

$$f_{\overrightarrow{L_p}}(\eta_{\text{paths}}) = N_{\text{old}} \int \exp\left[-\frac{1}{2\sigma^2}(\vec{\ell} - \vec{\mu}_\ell)^{\mathrm{T}}[\mathbf{P},\mathbf{P}](\vec{\ell} - \vec{\mu}_\ell)\right] d\vec{\ell} \tag{S26}$$

$$= N_{\text{old}} N_{\text{new}} \int \frac{\exp\left[\frac{-1}{2\sigma^2}(\vec{\ell} - \vec{\mu}_\ell)^{\mathrm{T}}[\mathbf{P},\mathbf{P}](\vec{\ell} - \vec{\mu}_\ell)\right]}{\sqrt{(2\pi\sigma^2)^{n_s}|[\mathbf{P},\mathbf{P}]^{-1}|}} d\vec{\ell} \tag{S27}$$

$$= N_{\text{old}} N_{\text{new}}. \tag{S28}$$

We also rewrite Equation S11 as

$$f_{\overrightarrow{L_p}}(\mathbf{P}\vec{\ell}) = N_{\text{old}} \exp\left[-\frac{1}{2\sigma^2}(\vec{\ell} - \vec{\mu}_\ell)^{\mathrm{T}}[\mathbf{P},\mathbf{P}](\vec{\ell} - \vec{\mu}_\ell)\right]. \tag{S29}$$

Dividing numerator by denominator, the distribution of the path locations after conditioning on the loops (equivalently, paths) meeting is

$$f_{\overrightarrow{L_p}|\eta_{\text{loops}}}(\vec{\ell_p}) = \frac{f_{\overrightarrow{L_p}}(\mathbf{P}\vec{\ell} \cap \eta_{\text{paths}})}{f_{\overrightarrow{L_p}}(\eta_{\text{paths}})} \tag{S30}$$

$$= \frac{N_{\text{old}} \exp\left[-\frac{1}{2\sigma^2}(\vec{\ell} - \vec{\mu}_\ell)^{\mathrm{T}}[\mathbf{P},\mathbf{P}](\vec{\ell} - \vec{\mu}_\ell)\right]}{N_{\text{old}} N_{\text{new}}} \tag{S31}$$

$$= \frac{\exp\left[-\frac{1}{2\sigma^2}(\vec{\ell} - \vec{\mu}_\ell)^{\mathrm{T}}[\mathbf{P},\mathbf{P}](\vec{\ell} - \vec{\mu}_\ell)\right]}{N_{\text{new}}}. \tag{S32}$$

This is a multivariate normal distribution with mean $\vec{\mu}_\ell = [\mathbf{P},\mathbf{P}]^{-1}[\mathbf{P},\mathbf{R}]\vec{\mu}$ and covariance $\sigma^2\mathbf{S} = \sigma^2[\mathbf{P},\mathbf{P}]^{-1}$. This is the likelihood of sample locations, $\overrightarrow{L}$, given Brownian motion down the ARG.

To derive the maximum likelihood parameter estimates, note that the log likelihood function for the parameters is given by

$$\log L(\vec{\mu}, \sigma^2) = -\frac{1}{2\sigma^2}(\vec{\ell} - \vec{\mu}_\ell)^{\mathrm{T}}[\mathbf{P},\mathbf{P}](\vec{\ell} - \vec{\mu}_\ell) - n_s \log\sigma + \text{const.} \tag{S33}$$

$$= -\frac{1}{2\sigma^2}\left[\vec{\ell}^{\mathrm{T}}[\mathbf{P},\mathbf{P}]\vec{\ell} - 2\vec{\mu}_\ell^{\mathrm{T}}[\mathbf{P},\mathbf{P}]\vec{\ell} + \vec{\mu}_\ell^{\mathrm{T}}[\mathbf{P},\mathbf{P}]\vec{\mu}_\ell\right] - n_s \log\sigma + \text{const.} \tag{S34}$$

We can use this to find the maximum likelihood root locations by first differentiating the log likelihood function with respect to each root location

$$
\begin{aligned}
-2\sigma^2 \frac{\partial \log L}{\partial \mu_i} &= -2\frac{\partial \vec{\mu}_\ell^{\mathrm{T}}}{\partial \mu_i}[\mathbf{P},\mathbf{P}]\vec{\ell} + \frac{\partial \vec{\mu}_\ell^{\mathrm{T}}[\mathbf{P},\mathbf{P}]\vec{\mu}_\ell}{\partial \mu_i} && \text{(S35)}\\
&= -2\frac{\partial \vec{\mu}_\ell^{\mathrm{T}}}{\partial \mu_i}[\mathbf{P},\mathbf{P}]\vec{\ell} + \frac{\partial \vec{\mu}_\ell^{\mathrm{T}}}{\partial \mu_i}[\mathbf{P},\mathbf{P}]\vec{\mu}_\ell + \vec{\mu}_\ell^{\mathrm{T}}[\mathbf{P},\mathbf{P}]\frac{\partial \vec{\mu}_\ell}{\partial \mu_i} && \text{(S36)}\\
&= -2\frac{\partial \vec{\mu}_\ell^{\mathrm{T}}}{\partial \mu_i}[\mathbf{P},\mathbf{P}]\vec{\ell} + 2\frac{\partial \vec{\mu}_\ell^{\mathrm{T}}}{\partial \mu_i}[\mathbf{P},\mathbf{P}]\vec{\mu}_\ell. && \text{(S37)}
\end{aligned}
$$

Now, since $\vec{\mu}_\ell = [\mathbf{P},\mathbf{P}]^{-1}[\mathbf{P},\mathbf{R}]\vec{\mu}$ then $\frac{\partial \vec{\mu}_\ell}{\partial \mu_i} = [\mathbf{P},\mathbf{P}]^{-1}[\mathbf{P},\mathbf{R}]\vec{e_i}$, where $\vec{e_i}$ is the unit vector of length n_s with 1 in the ith position. Therefore

$$
-2\sigma^2 \frac{\partial \log L}{\partial \mu_i} = -2\vec{e_i}^{\mathrm{T}}[\mathbf{R},\mathbf{P}]\vec{\ell} + 2\vec{e_i}^{\mathrm{T}}[\mathbf{R},\mathbf{P}][\mathbf{P},\mathbf{P}]^{-1}[\mathbf{P},\mathbf{R}]\vec{\mu}. \quad \text{(S38)}
$$

Setting the left hand side to zero for all $i \in \{1, 2, ..., r\}$ we get the maximum likelihood root locations, $\widehat{\vec{\mu}}$, as the solutions to a system of linear equations,

$$
\begin{aligned}
[\mathbf{R},\mathbf{P}][\mathbf{P},\mathbf{P}]^{-1}[\mathbf{P},\mathbf{R}]\vec{\mu} &= [\mathbf{R},\mathbf{P}]\vec{\ell} && \text{(S39)}\\
\Rightarrow \widehat{\vec{\mu}} &= \left([\mathbf{R},\mathbf{P}][\mathbf{P},\mathbf{P}]^{-1}[\mathbf{P},\mathbf{R}]\right)^{-1}[\mathbf{R},\mathbf{P}]\vec{\ell^*}. && \text{(S40)}
\end{aligned}
$$

Note that using this equation to get the root locations leads to unexpected behavior. Specifically, ancestor locations rapidly move away from each other as we go back in time (Figure S2). This is probably because, forwards in time, two Brownian motions that start at different locations have the highest probability of meeting in the middle, which forces them to diverge backwards in time. To avoid this issue, we use the unconditional distribution of $\overrightarrow{L_p}$ (Eq. 2, i.e., not conditioning on the paths meeting at the samples) to compute the maximum likelihood root locations,

$$
\widehat{\vec{\mu}} = (\mathbf{R}^{\mathrm{T}}\mathbf{S}_p^{g-}\mathbf{R})^{-1}\mathbf{R}\mathbf{S}_p^{g-}\mathbf{P}\vec{\ell^*}. \quad \text{(S41)}
$$

This behaves as we expected, with ancestor locations that do not diverge as strongly back in time (Figure S2), and we therefore use this method in the main text. We leave a more complete understanding of why the conditioned distribution behaves unexpectedly to future work. Note that when there is a single root the conditional and unconditional maximum likelihood root locations are equal (Figure S2) and collapse to our previously calculated MLE (Eq. S10).

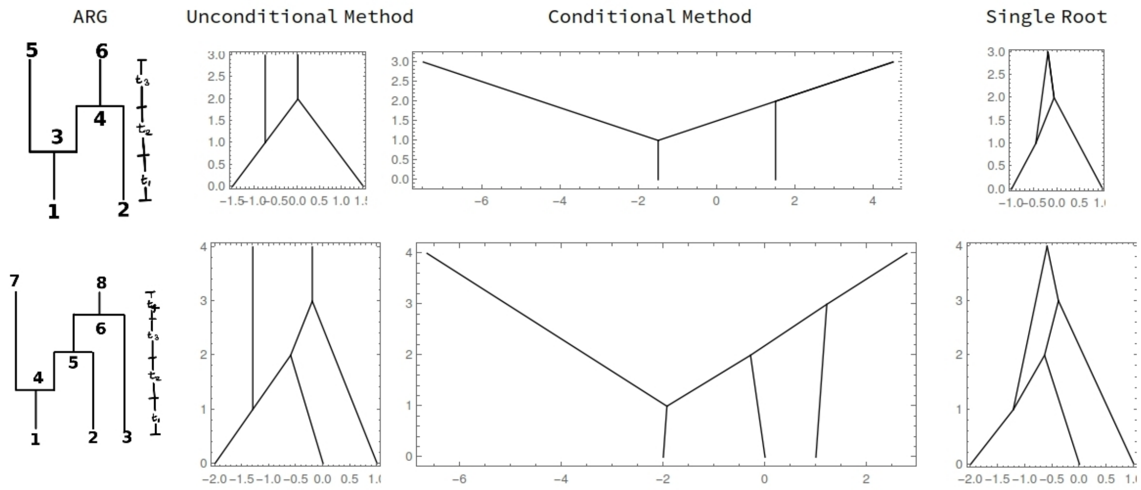

Figure S2: **Conditional vs. unconditional ancestor locations.** Inferred ancestor locations for two different ARGs (rows) with two different methods: unconditional (roots located with Equation S41) and conditional (roots located with Equation S39). When there is a single root (rightmost panel) the two methods converge.

Finally, differentiating the log likelihood (Equation S34) with respect to σ^2 and setting to zero, the maximum likelihood dispersal rate when there are multiple roots is

$$\widehat{\sigma^2} = \frac{(\vec{\ell^*} - \widehat{\vec{\mu}_\ell})^{\mathrm{T}} \mathbf{S}^{-1} (\vec{\ell^*} - \widehat{\vec{\mu}_\ell})}{n_s} \tag{S42}$$

$$= \frac{(\vec{\ell^*} - \widehat{\vec{\mu}_\ell})^{\mathrm{T}} \mathbf{P} \mathbf{S}_p^{g-} \mathbf{P} (\vec{\ell^*} - \widehat{\vec{\mu}_\ell})}{n_s}. \tag{S43}$$

S2 Equivalence of loop and path conditions

Here we prove that the loop and path conditions are equal, $\eta_{\text{loops}} = \eta_{\text{paths}}$, for any ARG. Before providing a formal proof, which requires more detailed notations, we first outline the idea:

1. To prove the equivalence we need to show that for each condition in η_{loops} there exists an equivalent condition or set of conditions in η_{paths} and vice versa. In other words, for each loop we need to find a pair of paths that only differ inside that loop. And conversely, for every pair of paths from the same sample, we need to find a set of loops such that any difference in the paths belongs to one of the loops.

2. Given a loop, we find a pair of paths as follows:

   (a) Find the bottom (more recent) and top (more ancestral) of the loop.

   (b) Find a path from the bottom to one of the samples.

   (c) Find a path from the top of the loop to one of the roots.

   (d) To get the two paths, insert each of the two paths around the loop in between the two paths found above.

3. Given a pair of paths ending at the same sample, we find a set of loops as follows:

   (a) Start from the sample and move up one node at a time until you hit a node that is not in one of the two paths. The previous node is the bottom of the first loop.

   (b) Now, find the first node above the bottom of the first loop that is common to the two paths. This is the top of the first loop.

   (c) If the two paths are identical above the top of the first loop, then we have found the equivalent loop condition.

   (d) If not, repeat the steps above starting from the top of the first loop to find the next loop and so on.

S2.1 Formal notation

Definition S2.1 (Directed graphs)**.** *A directed graph, G_d, is a two-tuple, (V, E_d), where V is the finite set of vertices/nodes and $E_d \subseteq V \times V$ is the set of edges.*

NOTE S2.1. *G_d is a directed graph so an edge from node v to node w, $(v, w) \in E_d$, does not necessarily imply an edge from node w to node v, $(w, v) \in E_d$. Given an edge (v, w), we call v the parent node and w the child node. Therefore, edges are directed from a parent node to a child node.*

Definition S2.2 (Parents)**.** *Given a directed graph, $G_d = (V, E_d)$, with node $v \in V$, then ch(v) $= \{w \in V : (v, w) \in E_d\}$ is the set of child nodes of v and par(v) $= \{w \in V : (w, v) \in E_d\}$ is the set of parent nodes of v. Further, $|par(v)|$ and $|ch(v)|$ denote the number of parent nodes and child nodes of v.*

Definition S2.3 (Paths)**.** *Given a directed graph, $G_d = (V, E_d)$, with two nodes $v, w \in V$, a path from v to w is a sequence of vertices $p = (v_0, v_1, ..., v_n)$ such that*

$(v_i, v_{i+1}) \in E_d\ \forall\ i \in \{0, 1, .., n-1\}$ and $v_0 = v$ and $v_n = w$. We will say v is connected to w, denoted by $v \to w$, if there exists a path from v to w. Further, we define for any $0 < l < m < n$, $p|_{v_l}^{v_m} = (v_l, v_{l+1}, ..., v_{m-1}, v_m)$, the section of path p between v_l and v_m.

Definition S2.4 (Loops)**.** *Given a directed graph, G_d, with two nodes $v, w \in V$, we say there is a loop between v and w if there exists two paths, $\lambda_L = (\lambda_{L0}, \lambda_{L1}, .., \lambda_{Ln_L})$ and $\lambda_2 = (\lambda_{R0}, \lambda_{R1}, .., \lambda_{Rn_R})$, from v to w such that $\lambda_{Li} \neq \lambda_{Rj}\ \forall\ i \in \{1, 2, .., n-1\}$ and $j \in \{1, 2, ..., n_2 - 1\}$, where $\lambda_{L0} = \lambda_{R0} = v$ and $\lambda_{Ln_L} = \lambda_{Rn_R} = w$. We will denote a loop by $\lambda = (\lambda_L, \lambda_R)$.*

Definition S2.5 (Ancestral recombination graph)**.** *An ancestral recombination graph (ARG) on a set of samples S is a 2-tuple, (G_d, t), where $G_d = (V, E_d)$ is a directed graph where $S \subsetneq V$, and $t : V \to \mathbb{R}^{\geq 0}$ is a function associating each node with its time such that*

1. $(v, w) \in E_d \Rightarrow t(v) < t(w)$
2. *ch(s)* $= \varnothing\ \forall\ s \in S$ and $|ch(v)| > 0\ \forall\ v \notin S$
3. $\exists\ v_{GMRCA} = \min_{t(v)}\{v \in V : v \to w\ \forall\ w \in V/\{v\}$ and $t(v) < t(w)\ \}$. v_{GMRCA} *is called the grand most recent common ancestor (GMRCA).*

We say $v \in V$ is a recombination node if $|par(v)| = 2$ and v is said to be a coalescence node if $|ch(v)| > 1$.

Definition S2.6 (SpARG)**.** *A d-dimensional spatial ancestral recombination graph, SpARG, is an ARG and a function $l : V \to \mathbb{R}^d$ which maps each vertex to its spatial location.*

S2.2 Spatial ancestral recombination graphs

We are interested in estimating the dispersal rate given a particular SpARG under a model of Brownian motion. We start by assuming that displacement along any given edge of an ARG is determined by an independent Brownian motion. We then condition on these independent Brownian motions forming the loops present in the ARG. For this we build a few more notations and definitions.

For any edge $(v, w) \in E_d$, let $B_{vw} \sim \mathcal{N}(0, \sigma^2 t_{vw})$ be the random displacement along that edge under Brownian motion, where $t_{vw} = t_v - t_w$ is the time-length of the

edge. We then define the displacement function, which takes a path $p \in P$ as input and returns the displacement,

$$\begin{aligned} D : P &\rightarrow \mathbb{R} \\ p = (v_i)_{i=0}^{k} &\mapsto \sum_{i=0}^{k-1} B_{v_i v_{i+1}} \end{aligned}$$

In order for these independent Brownian motions to form the loops in the ARG we need

$$\eta_{\text{loops}} = \{D(\lambda_1) = D(\lambda_2) : \lambda = (\lambda_1, \lambda_2) \text{ is a loop in the ARG }\}$$

Now, let X_i denote the displacement of the ith sample in S, s_i, relative to the GMRCA, v_{GMRCA}. Then the probability distribution of $\mathbf{X} = \{X_i\}_{i=1}^{n}$, where $n = |S|$ is the number of samples, is given by

$$P_{\mathbf{X}}(x_1, x_2, .., x_n) = p_{\mathbf{B}}(x_1, x_2, .., x_n | \eta_{\text{loops}}) \tag{S44}$$

where $B_i = D(p_i)$ is the random variable for the displacement along a path p_i and $\mathbf{B} = \{B_i\}_{i=1}^{n}$.

For Equation S44 to be well defined, i.e., for it to give a single value for a given set of inputs, the value should not depend on the choice of the path from a sample to the GMRCA. To define this more formally, let $P_i = \{(v_{ij})_{j=0}^{n_i} : v_{i0} = v_{\text{GMRCA}}, v_{in_i} = s_i, (v_{ij}, v_{i,j+1}) \in E_d \; \forall \; 0 \leq j < n_i\}$ be the set of paths from the GMRCA to the sample s_i. Now, we force the displacements along each path from the GMRCA to a given sample to be equal and call these set of conditions for all samples together as the path condition,

$$\begin{aligned} \eta_{i,\text{paths}} &= \{D(p_i^{(1)}) = D(p_i^{(2)}) : p_i^{(1)}, p_i^{(2)} \in P_i\} \\ \eta_{\text{paths}} &= \bigcup_{i=1}^{n} \eta_{i,paths} \end{aligned}$$

Now, as long as η_{paths} is true Equation S44 is well defined. We next show that $\eta_{\text{loops}} = \eta_{\text{paths}}$, which ensures Equation S44 is always well defined.

Lemma S2.1. $\eta_{paths} = \eta_{loops}$

Proof : [$\Rightarrow$] We will first show that $\eta_{\text{paths}} \subseteq \eta_{\text{loops}}$. Therefore, we need to show that given any two paths $p_i^{(1)} = (v_{ij}^{(1)})_{j=0}^{n_i^{(1)}}$ and $p_i^{(2)} = (v_{ij}^{(2)})_{j=0}^{n_i^{(2)}}$ from the GMRCA to a

sample s_i, there exists loops $\lambda^{(1)}, \lambda^{(2)}, ..., \lambda^{(m)}$ such that

$$D(\lambda_L^{(l)}) = D(\lambda_R^{(l)}) \ \forall\ 1 \leq l \leq m \Leftrightarrow D(p_i^{(1)}) = D(p_i^{(2)}).$$

Here is how to find the loops starting from the two distinct paths. Let $n_{\min} = \min\{n_i^{(1)}, n_i^{(2)}\}$ and $n_{\max} = \max\{n_i^{(1)}, n_i^{(2)}\}$. Then define $J := \{j \in \{0, 1, ..., n_{\min}\} : v_{il}^{(1)} = v_{il}^{(2)} \ \forall\ l \leq j\}$ and $j_{\text{st}} := \max J$. Therefore, j_{st} is the first node after which two paths start to diverge. Now, $j_{\text{st}} < n_{\max}$, otherwise we will have that $v_{il}^{(k_1)} = v_{il}^{(k_2)}$ for all l, which would mean the two paths are identical leading to a contradiction since we started with two distinct paths. Let $v_{\text{st}}^{(1)} = v_{ij_{\text{st}}}^{(1)} = v_{ij_{\text{st}}}^{(2)}$. This is the start of the first loop. To find the end of this loop, let $V_{\text{end}} := \{u \in p_i^{(1)} \cap p_i^{(2)} : t_u < t_{v_{\text{st}}^{(1)}}\}$. Then, the end of the loop is $v_{\text{end}}^{(1)} = \max\limits_{t(u)} V_{\text{end}}$. Note that $\lambda^{(1)} = (p_i^{(1)}|_{v_{\text{st}}^{(1)}}^{v_{\text{end}}^{(1)}}, p_i^{(2)}|_{v_{\text{st}}^{(1)}}^{v_{\text{end}}^{(1)}})$ is a loop.

Now, if $p_i^{(1)}|_{v_{\text{end}}^{(1)}}^{s_i} = p_i^{(2)}|_{v_{\text{end}}^{(1)}}^{s_i}$, then we are done. Since everything before $v_{\text{st}}^{(1)}$ and after $v_{\text{end}}^{(1)}$ are identical in the two paths, the displacement along the two paths being equal is the same as the displacements along the two sides of the loop $\lambda^{(1)}$ being equal.

If $p_i^{(1)}|_{v_{\text{end}}^{(1)}}^{s_i} \neq p_i^{(2)}|_{v_{\text{end}}^{(1)}}^{s_i}$, then we can repeat the above steps on $p_i^{(1)}|_{v_{\text{end}}^{(1)}}^{s_i}$ and $p_i^{(2)}|_{v_{\text{end}}^{(1)}}^{s_i}$, to find $v_{\text{st}}^{(2)}$ and $v_{\text{end}}^{(2)}$ such that $p_i^{(1)}|_{v_{\text{end}}^{(1)}}^{v_{\text{st}}^{(2)}} = p_i^{(2)}|_{v_{\text{end}}^{(1)}}^{v_{\text{st}}^{(2)}}$ and $\lambda^{(2)} = (p_i^{(1)}|_{v_{\text{st}}^{(2)}}^{v_{\text{end}}^{(2)}}, p_i^{(2)}|_{v_{\text{st}}^{(2)}}^{v_{\text{end}}^{(2)}})$ is a loop. Keep repeating this until we have $(v_{\text{st}}^{(k)}, v_{\text{end}}^{(k)})_{k=1}^m$ such that $\lambda^{(l)} = (p_i^{(1)}|_{v_{\text{st}}^{(l)}}^{v_{\text{end}}^{(l)}}, p_i^{(2)}|_{v_{\text{st}}^{(l)}}^{v_{\text{end}}^{(l)}})$ are loops and $p_i^{(1)}|_{v_{\text{end}}^{(l)}}^{v_{\text{st}}^{(l+1)}} = p_i^{(2)}|_{v_{\text{end}}^{(l)}}^{v_{\text{st}}^{(l+1)}} \ \forall\ 1 \leq l \leq m$ where $v_{\text{st}}^{(m+1)} = s_i$. We can do this because it is a finite graph.

Therefore, the displacement along the two parts being equal is equivalent to the displacements forming the loops $\lambda^{(1)}, \lambda^{(2)}, .., \lambda^{(m)}$. Thus, $\eta_{\text{paths}} \subseteq \eta_{\text{loops}}$.

[$\Rightarrow$] Now we will show that $\eta_{\text{loops}} \subseteq \eta_{\text{paths}}$. That is, we show that given a loop λ, there exists and a pair of paths $p_i^{(1)}$ and $p_i^{(2)}$ from the GMRCA to a sample s_i such that

$$D(p_i^{(1)}) = D(p_i^{(2)}) \Leftrightarrow D(\lambda_1) = D(\lambda_2).$$

You find two distinct paths given a loop λ in the following way. Suppose λ is a loop from v to w. By definition of an ARG, $v_{\text{GMRCA}} \to v$ Let this path be $p_{v_{\text{GMRCA}} \to v}$. Now, if $w \in S$, then $s_i = w$ and $p_i^{(1)} = p_{v_{\text{GMRCA}} \to v} \cup \lambda_1$ and $p_i^{(2)} = p_{v_{\text{GMRCA}} \to v} \cup \lambda_2$ are two distinct paths from the GMRCA to the sample. Therefore, the condition for the Brownian motions to form the loop λ_1 is the same as the displacement along $p_i^{(1)}$ being equal to $p_i^{(2)}$.

If $w \notin S$, then we claim that there exists a sample $s_i \in S$ such that $w \to s_i$. Suppose not, i.e., $w \nrightarrow s_i \ \forall \ 1 \leq i \leq n$. Since $w \notin S$, therefore $\exists \ w^{(1)}$ such that $(w, w^{(1)}) \in E_d$ by definition of an ARG. Now $w^{(1)}$ also does not belong to S, otherwise we will have a vertex in S that is connected to w. Similarly, by induction we can construct $\{w^{(k)}\}_{k \in \mathbb{N}}$ such that $(w^{(k)}, w^{(k+1)}) \in E_d$ and $w^{(k)} \notin S \ \forall \ k \in \mathbb{N}$. Therefore we have infinite vertices in the *ARG* which is a contradiction. Therefore our claim has to be true. Let the path from w to s_i be $p_{w \to s_i}$, then $p_{v_{\text{GMRCA}} \to v} \cup \lambda_1 \cup p_{w \to s_i}$ and $p_{v_{\text{GMRCA}} \to v} \cup \lambda_2 \cup p_{w \to s_i}$ are the two required paths. Therefore, $\eta_{\text{loops}} \subseteq \eta_{\text{paths}}$.

Therefore, we have that $\eta_{\text{loops}} = \eta_{\text{paths}}$. Q.E.D

S3 Minimal path matrix

The set of loop conditions, η_{loops}, will have exactly as many conditions as the number of recombination nodes, say k, in the ARG. However, the size of the full paths matrix is n_p, the total number of paths, which is greater than or equal to $k + n_s$ (e.g., if each loop is placed alone on a terminal branch) and is bounded above by $2^k n_s$ (e.g., if all loops are placed on the branch above the GMRCA). The number of conditions in η_{paths} is bounded by $\binom{n_p}{2}$, which therefore increases at least quadratically in k and potentially exponentially. Therefore, η_{paths} has multiple redundant conditions. We only need one pair of paths for each condition η_{loops}, which only differ in one loop. We also need at least one path to each sample. Therefore, if chosen correctly, we only need $n_s + k$ paths to calculate the correct estimates. We call the matrix of shared times of these $n_s + k$ paths the "minimal path matrix".

Though there are existing methods in `Python` (using the `all_simple_paths()` function of `networkx` package) to identify all paths from the roots to the samples, calculating the intersection between these paths does not scale well to larger ARGs, primarily due to repeated calculation of common edges across different paths. We therefore developed an algorithm, outlined below, that requires traversing each edge only once and, in doing so, have greatly sped up the calculation of $\mathbf{S}_p$.

Briefly, the algorithm entails a bottom-up traversal of the ARG starting at the sample nodes and updating the shared time matrix as we move upwards towards the roots (Figure S3). For each coalescent node visited, the algorithm calculates the edge length between that node and its parent. This is added to the corresponding cells in the shared time matrix. In addition, when we reach a recombination node (which has multiple parents), the relevant row and column are duplicated, expanding the size of

the matrix and corresponding with the separation of these paths in the ARG. This keeps the size of the matrix small for as long as possible, making it more efficient. We then add the edge length to each parent in their respective paths. Currently, the algorithm is implemented using the `tskit` package (Kelleher *et al.*, 2018).

S3.1 Algorithm

1. Initialization

    - The shared time matrix $\mathbf{S} \leftarrow [0]_{n_s \times n_s}$, a zero square matrix of size n_s, the number of samples. The entry of the ith row and j^{th} column is denoted by s_{ij}.
    - The list of paths $PL \leftarrow [[1], [2], ..., [n_s]]$ with one path for each sample node.

2. Loop through every node in the ARG in time ascending order. Let u be the focal node. Let I_u be the set of indices of the paths in PL that currently end in u. Let k_u be the number of parent nodes. Then for each node u,

    (a) If $k_u = 0$, u is the root and the loop ends.

    (b) If $k_u = 1$, with parent node v, then do the following:

    - $s_{ij} \leftarrow s_{ij} + t_{uv}$ for all i, j in I_u, where t_{uv} is the length of edge (v, u). Add shared time along edge to appropriate covariance terms.
    - $PL[i] \leftarrow PL[i] + [v]$ for all $i \in I_u$. Extend all paths that currently end at u to v.

    (c) If $k_u = 2$, with parent nodes v_1 and v_2, then do the following:

    - Pick one index from I_u, say l.
    - $PL \leftarrow PL + [PL[l]]$. Duplicate the lth path. Don't update I_u.
    - $PL[i] \leftarrow PL[i] + [v_1]$ for all i in I_u. Extend all existing paths that end at u to v_1.
    - $PL[-1] \leftarrow PL[-1] + [v_2]$. Extend the new path formed in this step to v_2.
    - $\mathbf{S} \leftarrow \begin{bmatrix} \mathbf{S} & \mathbf{S}[:][l] \end{bmatrix}$. Duplicate the lth column of $\mathbf{S}$.
    - $\mathbf{S} \leftarrow \begin{bmatrix} \mathbf{S} \\ \mathbf{S}[:][l] \end{bmatrix}$. Duplicate the lth row of $\mathbf{S}$.
    - $s_{ij} \leftarrow s_{ij} + t_{uv_1}$ for all i, j in I_u.

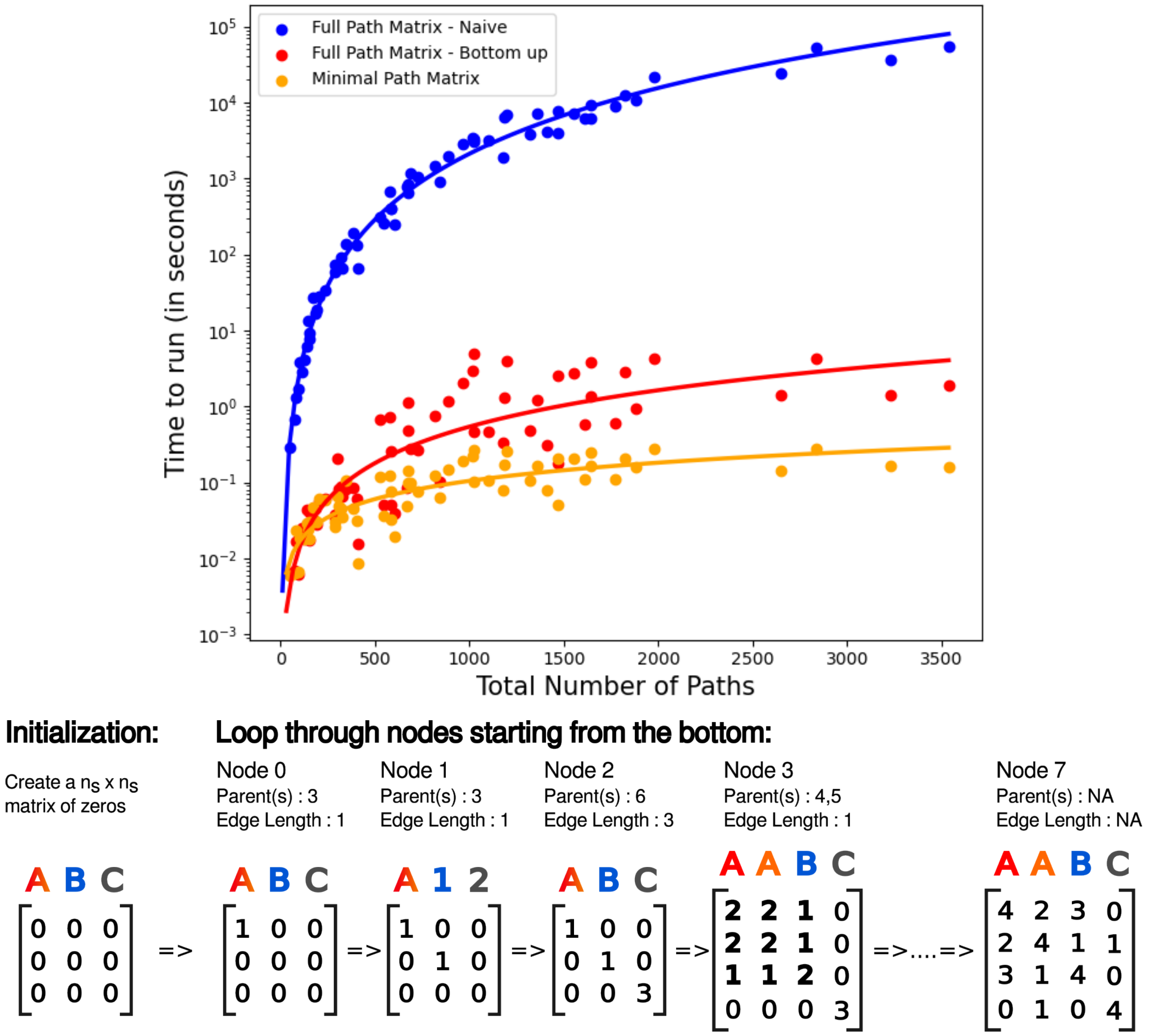

Figure S3: **Algorithm and its benchmarks**. (top) Number of seconds to compute the full path and minimal path matrices using different algorithms as a function of the total number of paths in the ARG. "Full Path Matrix - Naive" (blue) uses existing `Python` methods to compute the full path matrix. "Full Path Matrix - Bottom up" (red) instead computes the full path matrix with a single bottom-up traversal of the ARG. "Minimal Path Matrix" (orange) uses the bottom-up method to compute the path matrix for the smallest set of linearly independent paths, which is sufficient for estimating parameters of interest. Random ARGs of various sizes were generated (number of samples ranged up to 500, sequence lengths up to 5000 basepairs with recombination rate 10^{-8}) using `msprime` (Baumdicker *et al.*, 2022). The solid lines are the best fits under a power law. The best fit exponents for the power law are 2.946 (Full Path Matrix - Naive), 1.432 (Full Path Matrix - Bottom up) and 0.853 (Minimal Path Matrix). (bottom) Steps of our algorithm for the ARG in Figure 1.

- $s_{ll} \leftarrow s_{ll} + t_{uv_2}$.

3. The end result is **S**, the minimal path matrix.

S4 Alternative models

Here we explore the dispersal estimates from two alternative models: (a) the relaxed meeting model and (b) the windowing approach. The dispersal estimates from both models are shown as a function of the number of trees used in the partial ARG in Figure S4. We briefly describe the two models and the behavior of their dispersal estimates below.

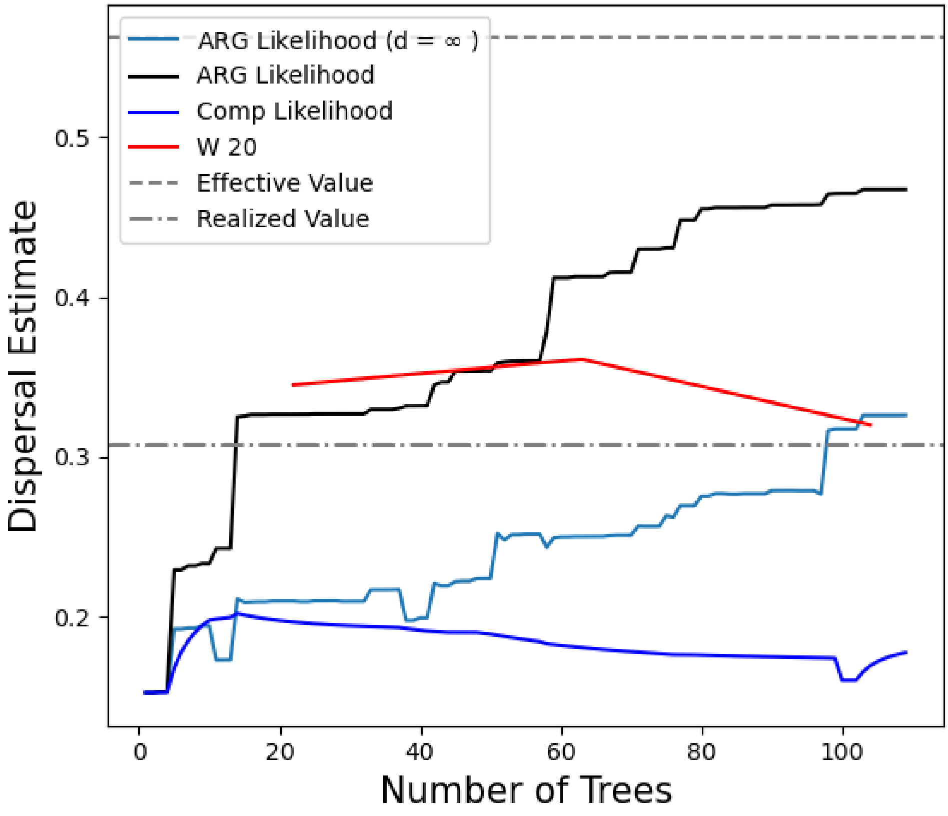

Figure S4: **Dispersal estimates under alternative models**. Dispersal rate computed from an ARG with 10 samples under different methods as a function of the number of trees. "ARG Likelihood (d = ∞)" is the dispersal estimate from the full ARG under the relaxed meeting model. "W 20" is the dispersal estimate from the windowing approach which uses a partial ARG with 20 tree on each side of the focal tree. All other methods are as in Figure 5.

S4.1 Relaxed meeting model

One alternative is the relaxed meeting model, which is identical to our primary model except the parents of a recombination node need not be close to one another in geographic space (here parents means the actual parents of the recombination node, one generation back, not the parent nodes of the recombination node in the ARG). The location of the recombination node is then taken to be average of its parent's locations. The sample matrix under this model, $\mathbf{S}_\infty$, has been computed in Bastide *et al.* (2018) (simply set $\gamma_e = 1/2$ in their model), which we refer to for more details. Here we show how $\mathbf{S}_\infty$ can be computed from the full path matrix, $\mathbf{S}_p$.

Let P_i be the set of paths from any one of the roots to sample s_i. Then the covariance between two samples s_i and s_j is (Bastide *et al.*, 2018)

$$\sigma^2 \sum_{p_i \in P_i} \sum_{p_j \in P_j} \frac{1}{2^{|k_i + k_j|}} \sum_{e \in p_i \cap p_j} t_e, \tag{S45}$$

where $p_i \cap p_j$ is the set of common edges between the two paths and k_i is the number of recombination nodes along path p_i. Let $\vec{W}$ be a $n_p \times 1$ vector which encodes the weights associated with each path. The lth entry of $\vec{W}$ is $\dfrac{1}{2^{k_l}}$. Then the sample matrix under this model is

$$\mathbf{S}_\infty = \mathbf{P}^T(\mathbf{S}_p \circ (\vec{W}\vec{W}^T))\mathbf{P}, \tag{S46}$$

where $\circ$ is the elementwise multiplication (Hadamard product) of the two matrices. We need to extend the Bastide *et al.* (2018) to incorporate multiple roots in order to estimate dispersal rates in chopped ARGs. With multiple roots the mean of a sample location is the weighted average of the locations of all the roots it is connected to, where the weight is $\frac{1}{2^{k_r}}$ and k_r is the number of recombination nodes along the path from root r. This is given by

$$\mathbf{R}\vec{\mu} = \mathbf{P}^T(\mathbf{R} \circ (\mathbb{1}_{n_r}^T \otimes \vec{W}))\vec{\mu}. \tag{S47}$$

Then the maximum likelihood estimates of the root locations and the dispersal rate are

$$\widehat{\vec{\mu}} = (\mathbf{R}^T\mathbf{S}_\infty^{-1}\mathbf{R})^{-1}\mathbf{R}^T\mathbf{S}_\infty^{-1}\vec{\ell^*} \tag{S48}$$

$$\widehat{\sigma^2} = \frac{(\vec{\ell^*} - \mathbf{R}\widehat{\vec{\mu}})^T\mathbf{S}_\infty^{-1}(\vec{\ell^*} - Rp\widehat{\vec{\mu}})}{n_s}. \tag{S49}$$

This dispersal rate estimate still increases with the number of trees (Figure S4) but the slope of increase is smaller than under our primary model and, further, we also see occasional declines. This emphasizes that the problem of loops has been reduced but not removed.

S4.2 Windowing approach

Another alternative is to take a windowing approach. Here we fix a window size, w, then build partial ARGs from disjoint groups of $2w$ trees (i.e., an ARG with trees 0 to $2w$, an ARG with trees $2w{+}1$ to $4w$, etc.). We then take the composite likelihood of dispersal over the partial ARGs. The maximum composite likelihood dispersal estimate is then average maximum likelihood estimate over partial ARGs. For a given window size, the dispersal rate does not monotonically increase as we include more partial ARGs (Figure S4). However, larger window sizes will give larger dispersal estimates and it is not clear how to choose a good window size for a given dataset.

S5 Recombination nodes increase clustering of sample locations under our model

Consider an ARG with n_s samples and n_t marginal trees. Now consider the partial ARG G_1 over the first $k < n_t$ trees, which has, say, n_p paths. Let the sample matrix for this partial ARG be $\mathbf{S}_1$ (of size $n_s \times n_s$) and the corresponding minimal path matrix be $\mathbf{S}_{p,1}$ (of size $n_s+k-1 \times n_s+k-1$). Next consider the partial ARG G_2 over the first $k+1$ trees. This will have n_p+1 paths. We call the corresponding sample and minimal path matrices $\mathbf{S}_2$ (also of size $n_s \times n_s$) and $\mathbf{S}_{p,2}$ (of size $n_s+k \times n_s+k$), respectively. We know from Equation 3 that

$$\mathbf{S}_1^{-1} = \mathbf{P}_1^T \mathbf{S}_{p,1}^{-1} \mathbf{P}_1 \quad \text{(S50)}$$

$$\mathbf{S}_2^{-1} = \mathbf{P}_2^T \mathbf{S}_{p,2}^{-1} \mathbf{P}_2. \quad \text{(S51)}$$

We want to show that the sample locations are more clustered for the partial ARG with more recombination nodes, G_2. Let $X_i^{(j)}$, $1 \le i \le n_s$, $j = 1, 2$, be the sample locations distributed with covariance matrix $\mathbf{S}_j$. We are interested in the variance among these which we depict by $V(\mathbf{S}_j)$. Since this a random variable we are

interested in its expectation $\mathbf{E}[V(\mathbf{S}_j)]$,

$$\begin{aligned} V(\mathbf{S}_j) &= \frac{1}{n_s}\sum_{i=1}^{n_s}(X_i^{(j)} - \frac{1}{n_s}\sum_{i=1}^{n_s} X_i^{(j)})^2 && \text{(S52)} \\ &= \frac{1}{n_s}\sum_{i=1}^{n_s}(X_i^{(j)})^2 - \Big(\frac{1}{n_s}\sum_{i=1}^{n_s} X_i^{(j)}\Big)^2 && \text{(S53)} \\ \mathbf{E}[V(\mathbf{S}_j)] &= \frac{1}{n_s}\sum_{i=1}^{n_s}\mathrm{Var}(X_i^{(j)}) - \frac{1}{n_s^2}\sum_{k,l=1}^{n_s}\mathrm{Cov}(X_k^{(j)}, X_l^{(j)}) && \text{(S54)} \\ &= \frac{1}{n_s}\mathrm{Tr}(\mathbf{S}_j) - \frac{1}{n_s^2}\mathbb{1}_{n_s}^T\mathbf{S}_j\mathbb{1}_{n_s}. && \text{(S55)} \end{aligned}$$

Therefore, we want to show that

$$\mathbf{E}[V(\mathbf{S}_1)] \geq \mathbf{E}[V(\mathbf{S}_2)]. \quad \text{(S56)}$$

To prove this we use two properties of the variance. First, it is additive, i.e., $\mathbf{E}[V(\mathbf{A}+\mathbf{B})] = \mathbf{E}[V(\mathbf{A})] + \mathbf{E}[V(\mathbf{B})]$ and second, it is positive, $\mathbf{E}[V(\mathbf{A})] > 0$, for any covariance matrix $\mathbf{A}$.

We can write $\mathbf{S}_{p,2}$ and $\mathbf{P}_2$ as an "extension" of $\mathbf{S}_{p,1}$ and $\mathbf{P}_1$, respectively. Specifically,

$$\begin{aligned} \mathbf{S}_{p,2} &= \begin{bmatrix} \mathbf{S}_{p,1} & \vec{v} \\ \vec{v}^T & t \end{bmatrix} && \text{(S57)} \\ \mathbf{P}_2 &= \begin{bmatrix} \mathbf{P}_1 \\ \vec{e}_z^T \end{bmatrix}, && \text{(S58)} \end{aligned}$$

where $\vec{v}$ is the shared time of the new path in the minimal path set of G_2 with all paths in the minimal path set of G_1, $\vec{e}_z$ is the unit vector with all 0s except 1 at the zth position, where z is the sample at which the new path ends, and t is the time from the root to the samples. Now, we can use block matrix inversion to relate $\mathbf{S}_{p,2}^{-1}$ and

$\mathbf{S}_{p,1}^{-1}$,

$$\begin{aligned}
\mathbf{S}_{p,2}^{-1} &= \begin{bmatrix} \mathbf{S}_{p,1} & \vec{v} \\ \vec{v}^T & t \end{bmatrix}^{-1} && \text{(S59)} \\
&= \begin{bmatrix} \mathbf{S}_{p,1}^{-1} + \frac{\mathbf{S}_{p,1}^{-1}\vec{v}\vec{v}^T\mathbf{S}_{p,1}^{-1}}{t-\vec{v}^T\mathbf{S}_{p,1}^{01}\vec{v}} & \frac{-\mathbf{S}_{p,1}^{-1}\vec{v}}{t-\vec{v}^T\mathbf{S}_{p,1}^{01}\vec{v}} \\ -\frac{\vec{v}^T\mathbf{S}_{p,1}^{-1}}{t-\vec{v}^T\mathbf{S}_{p,1}^{01}\vec{v}} & \frac{1}{t-\vec{v}^T\mathbf{S}_{p,1}^{01}\vec{v}} \end{bmatrix} && \text{(S60)} \\
&= \begin{bmatrix} \mathbf{S}_{p,1}^{-1} & 0 \\ 0 & 0 \end{bmatrix} + \frac{1}{t - \vec{v}^T\mathbf{S}_{p,1}^{01}\vec{v}} \begin{bmatrix} \mathbf{S}_{p,1}^{-1}\vec{v}\vec{v}^T\mathbf{S}_{p,1}^{-1} & -\mathbf{S}_{p,1}^{-1}\vec{v} \\ -\vec{v}^T\mathbf{S}_{p,1}^{-1} & 1 \end{bmatrix} && \text{(S61)} \\
&= \begin{bmatrix} \mathbf{S}_{p,1}^{-1} & 0 \\ 0 & 0 \end{bmatrix} + \mathbf{M}, && \text{(S62)}
\end{aligned}$$

where M is also positive semidefinite (it can be shown that $[\vec{x}^T \ x_0]^T M [\vec{x}^T \ x_0]^T = ||x_0 - \vec{x}^T \mathbf{S}_{p,1}^{-1}\vec{v}||_2^2 > 0$ for every vector $[\vec{x}^T \ x_0]^T$). We can then relate $\mathbf{S}_1$ and $\mathbf{S}_2$,

$$\begin{aligned}
\mathbf{S}_2^{-1} &= \mathbf{P}_2^T\mathbf{S}_{p,2}^{-1}\mathbf{P}_2 && \text{(S63)} \\
&= \mathbf{P}_1^T\mathbf{S}_{p,1}^{-1}\mathbf{P}_1 + \mathbf{P}_2^T M \mathbf{P}_2 && \text{(S64)} \\
&= \mathbf{S}_1^{-1} + \mathbf{M}_2, && \text{(S65)}
\end{aligned}$$

where $\mathbf{M}_2 = \mathbf{P}_2^T M \mathbf{P}_2$ is also positive semidefinite. Now, we multiply the whole equation by $\mathbf{S}_1$ on the left (and right respectively) and $\mathbf{S}_2$ on the right (and left respectively) to get

$$\begin{aligned}
\mathbf{S}_1 &= \mathbf{S}_2 + \mathbf{S}_1\mathbf{M}_2\mathbf{S}_2 && \text{(S66)} \\
\mathbf{S}_1 &= \mathbf{S}_2 + \mathbf{S}_2\mathbf{M}_2\mathbf{S}_1. && \text{(S67)}
\end{aligned}$$

Therefore, we have that $\mathbf{S}_1\mathbf{M}_2\mathbf{S}_2 = \mathbf{S}_2\mathbf{M}_2\mathbf{S}_1$. Lets call this matrix $\mathbf{M}_3$. $\mathbf{M}_3$ is symmetric ($\mathbf{M}_3^T = (\mathbf{S}_1\mathbf{M}_2\mathbf{S}_2)^T = \mathbf{S}_2^T\mathbf{M}_2^T\mathbf{S}_1^T = \mathbf{S}_2\mathbf{M}_2\mathbf{S}_1 = \mathbf{M}_3$) and the product of three positive semi-definite matrices. Therefore, $\mathbf{M}_3$ is positive semi-definite and hence a covariance matrix, which gives us

$$\begin{aligned}
\mathbf{E}[V(\mathbf{S}_1)] &= \mathbf{E}[V(\mathbf{S}_2)] + \mathbf{E}[V(\mathbf{M}_3)] && \text{(S68)} \\
&\geq \mathbf{E}[V(\mathbf{S}_2)], && \text{(S69)}
\end{aligned}$$

proving our statement.

We can potentially use Equation S65 to explicitly show that, for the same location

of samples $\vec{\ell}$, the dispersal estimate from the partial ARG with more recombination nodes, G_2, is greater. To see this note that

$$\begin{aligned}(\vec{\ell} - \mu \mathbb{1}_{n_s})^T \mathbf{S}_2^{-1} (\vec{\ell} - \mu \mathbb{1}_{n_s}) &= (\vec{\ell} - \mu \mathbb{1}_{n_s})^T \mathbf{S}_1^{-1} (\vec{\ell} - \mu \mathbb{1}_{n_s}) + (\vec{\ell} - \mu \mathbb{1}_{n_s})^T \mathbf{M}_2 (\vec{\ell} - \mu \mathbb{1}_{n_s}) \quad \text{(S70)} \\ &\geq (\vec{\ell} - \mu \mathbb{1}_{n_s})^T \mathbf{S}_1^{-1} (\vec{\ell} - \mu \mathbb{1}_{n_s}), \quad \text{(S71)}\end{aligned}$$

which would be a a complete proof if the root location estimate μ was the same for G_1 and G_2. Unfortunately that is not the case. Therefore, we need to prove some inequality regarding those to complete the proof, which at the moment remains elusive.

S6 Single tree, unbounded space

To verify that our method works under our model in the absence of recombination, we simulated a single tree in unbounded space for different dispersal rates. Our estimates closely track the simulated value (Figure S5), validating our method.

Further, note that for a tree, each sample has a unique path from the root associated with it. Therefore, we have,

$$\begin{aligned}\mathbf{S}_p &= \mathbf{S} \quad \text{(S72)} \\ \mathbf{P} &= \mathbf{I} \quad \text{(S73)}\end{aligned}$$

where $\mathbf{S}$ is the shared time between each pair of paths (sample lineages). Therefore, the dispersal estimate (Eq S9 and Eq S10) reduces to the well known estimates for Brownian motion on a tree,

$$\begin{aligned}\widehat{\mu} &= (\mathbb{1}_{n_s} \mathbf{S}^{-1} \mathbb{1}_{n_s})^{-1} \mathbb{1}_{n_s} \mathbf{S}^{-1} \vec{\ell^*} \quad \text{(S74)} \\ \widehat{\sigma^2} &= \frac{(\vec{\ell^*} - \mu \mathbb{1}_{n_s})^T \mathbf{S}^{-1} (\vec{\ell^*} - \mu \mathbb{1}_{n_s})}{n_s}, \quad \text{(S75)}\end{aligned}$$

S7 Boundary effects

To confirm that the reflecting boundaries in our simulations are not the main cause of the bias in location estimates, we ran the same simulations as used for Figure 6 but now in a larger area (90,000 square units versus the original 10,000 square units) but only sampled individuals from the center of the range. We expect that the

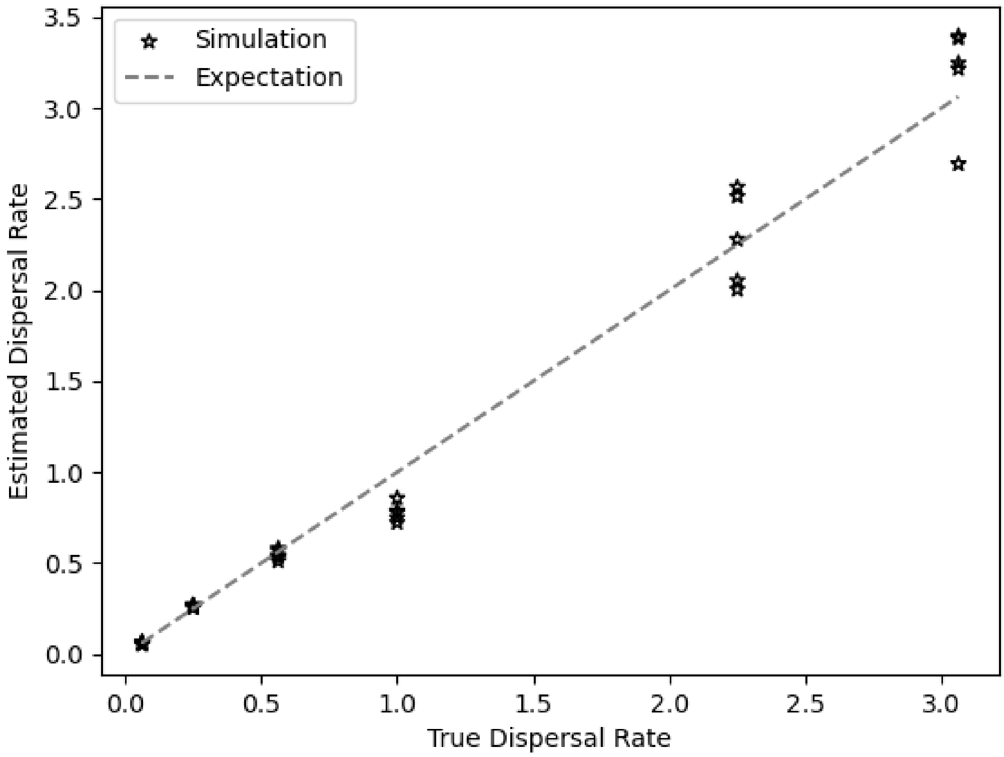

Figure S5: **Verification of our method**. Dispersal rate estimates for a tree simulated in unbounded space. Each point is a tree with 100 samples.

shared lineages of these samples have interacted very little with the boundaries of the simulation. Even still, we observe relatively similar patterns as previously (Figure S6), in particular, a center bias. Errors were higher in this modified simulation as ancestors were able to disperse outside of the sampled range.

S8 One-dimensional simulations

To confirm that the observed bias in our estimates is not due to a characteristic of Brownian motion in two dimensions, we reran simulations but now in one dimension. We kept the parameters consistent with the original two-dimensional simulations, but now used a simulated area that was 100 units long. Once again, we observed a higher location error when using an ARG versus the local tree, with a bias towards the center (Figure S7), and a dispersal rate that increases monotonically as more trees are included in the ARG (Figure S8).

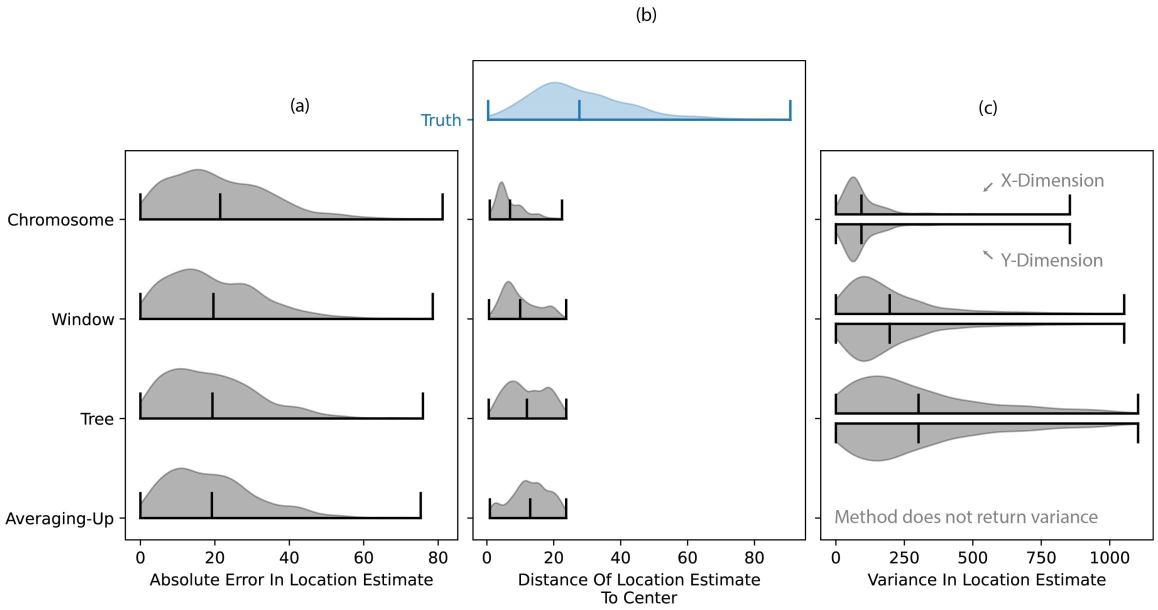

Figure S6: **Check for boundary effects**. Recreation of Figure 6 but with a modified simulation that included a larger area in which individuals could disperse. Individuals were only sampled from the center of the area; this was done to reduce any effects of the reflecting boundaries.

S9 Assessing estimated uncertainty in ancestor locations

Our ARG method can be overconfident in its estimates of ancestral locations. Using the simulations from Figure 6 and the true effective dispersal rate, we created a coverage plot (Figure S9), which shows what percentage of ancestors fall within the estimates' confidence intervals as the size of those intervals is increased. We did this using the full ARG ("Chromosome"), a window of 100 trees on either side of a focal tree ("Window"), and the focal tree ("Tree"), as in Figure 6. When using the local tree or a small window, the confidence intervals are too large. In contrast, when we use the full chromosome our confidence intervals are too small.

S10 Ancestor location error over time

Figure S10 shows the bias in location estimates (true - estimated) color-stamped by the time. We can see that true locations greater than 50 (center of habitat) have positive error while locations less than 50 have a negative error. This is the center

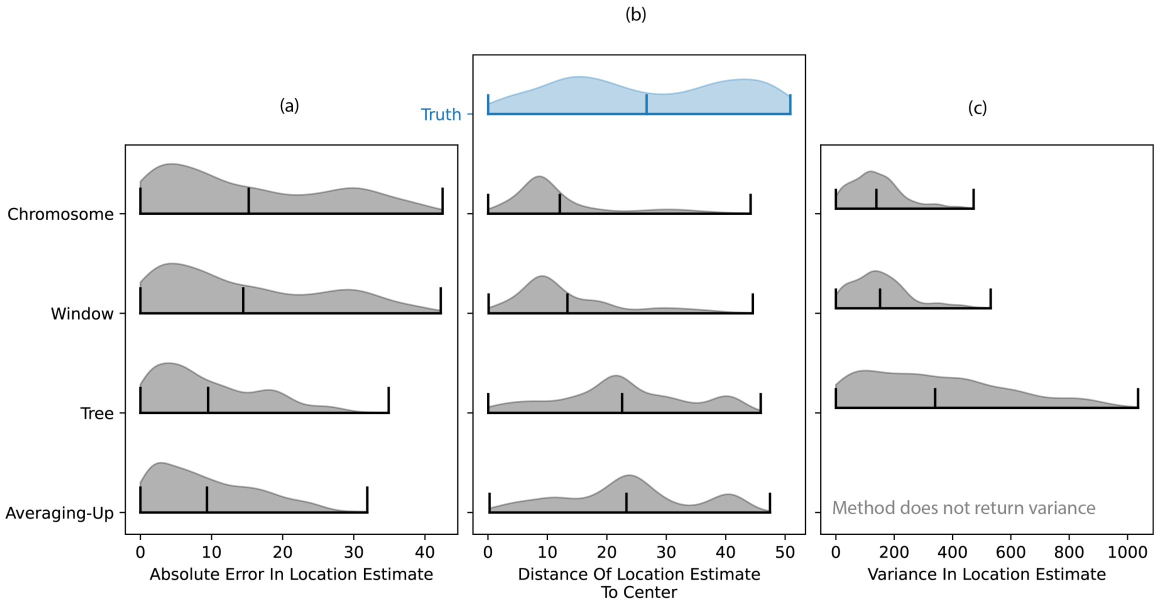

Figure S7: **Ancestor locations from one-dimensional simulations**. Recreation of Figure 6 but simulating in only one dimension. We only included 500 samples in this analysis as the population size in the one-dimensional simulation is smaller than in the two-dimensional simulation. The “Window” and “Chromosome” results are very similar here because we still used a window of 100 trees on either side of focal tree and this is relatively close to the number of trees along the chromosome.

bias described in the main text. This center-bias gets more severe as we go back in time and as we include more trees in the ARG.

S11 The problem of loops and a potential way forward

To compute maximum likelihoods estimates, we use the probability distribution of locations conditioned on lineages meeting at recombination nodes, i.e.,

$$p(\vec{\ell}\,|\eta_{paths}, \text{ARG}, \sigma^2, \mu) = \frac{p(\vec{\ell}\,, \eta_{paths}|\sigma^2, \mu, \text{ARG})}{p(\eta_{paths}|\sigma^2, \mu, \text{ARG})}, \tag{S76}$$

which is the probability distribution for $\overrightarrow{L}$. Alternatively, we could choose not to condition on lineages meeting and just use

$$p(\vec{\ell}\,, \eta_{paths}|\sigma^2, \mu, \text{ARG}). \tag{S77}$$

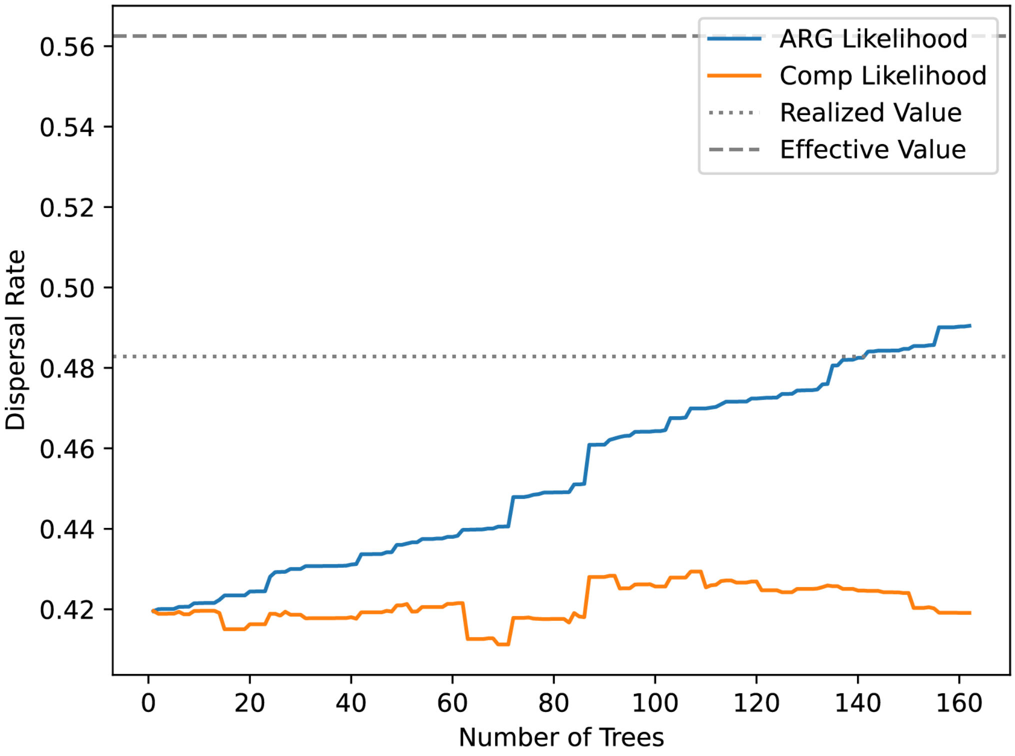

Figure S8: **Dispersal rate from one-dimensional simulations**. Recreation of Figure 5 but simulating in only one dimension (see Figure S7 for more details).

Now, the denominator was $p(\eta_{paths}|\sigma^2, \mu, \text{ARG}) = \frac{\sqrt{(2\pi\sigma^2)^{n_s}|\mathbf{S}|}}{\sqrt{(2\pi\sigma^2)^{\text{rnk } \mathbf{S}_p}|\mathbf{S}_p|}}$, where $\mathbf{S}_p$ is the shared times between all paths and $\mathbf{S}$ is the sample covariance matrix. This term depends on σ^2 and hence ignoring it alters the maximum likelihood estimate of σ^2. Using the unconditioned probability (Eq. S77), the dispersal estimate now converges with an increasing number of trees (Figure S11). However, the unconditioned probability is not a probability distribution for the observed locations. Further, the distribution of an internal node location, ℓ_a is given by $p(\ell_a|\vec{\ell}, \eta_{paths}, \hat{\sigma^2}, \hat{\mu})$. The unconditioned version of this, $p(\ell_a, \eta_{paths}|\vec{\ell}, \hat{\sigma^2}, \hat{\mu})$, is not a probability distribution for ℓ_a so there is no principled way of calculating the variance.

The conditioned distribution (Eq. S76) is the right quantity to consider given the model but model misspecification creates biased estimates. The unconditioned distribution (Eq. S77) is the wrong quantity but displays some better properties. This suggests that much of the model misspecification and resulting bias may come

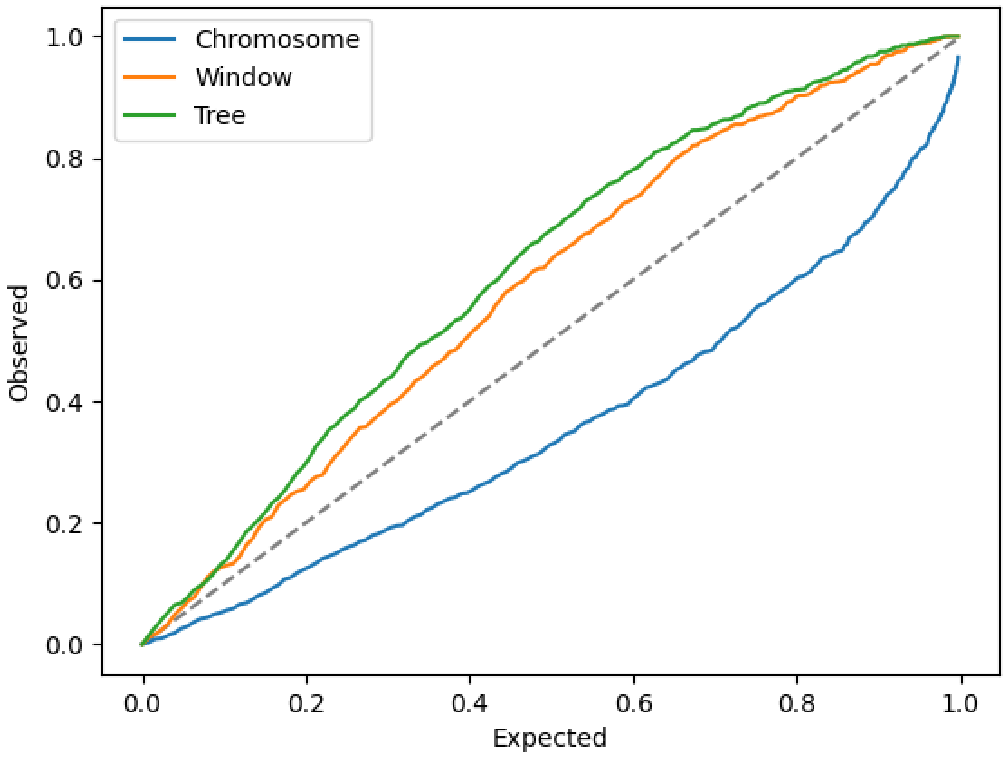

Figure S9: **Coverage of ancestor location confidence intervals**. We used the true effective dispersal rate to calculate the variance around each ancestral location estimate. We plot the observed fraction of ancestors that fall within an estimated confidence interval.

from conditioning on loops. To see this, let the probability distribution under the right model be $p_R(\vec{\ell}\,|\eta_{paths}, \text{ARG}, \sigma^2, \mu)$. We are using $p(\vec{\ell}\,|\eta_{paths}, \text{ARG}, \sigma^2, \mu)$ as an approximation. Since Brownian motion is a good approximation for the movement of individual lineages (e.g., on trees), the numerator $p(\vec{\ell}\,, \eta_{paths}|\text{ARG}, \sigma^2, \mu)$ is likely a good approximation for $p_R(\vec{\ell}\,, \eta_{paths}|\text{ARG}, \sigma^2, \mu)$. However, because lineages are so unlikely to meet under Brownian motion, the conditioning is too strong,

$$p(\eta_{paths}|\text{ARG}, \sigma^2, \mu) << p_R(\eta_{paths}|\text{ARG}, \sigma^2, \mu).$$

Therefore, the maximum likelihood estimates with the condition are too high and the estimates without the condition are too low (compared to $p_R(\vec{\ell}\,|\eta_{paths}, \text{ARG}, \sigma^2, \mu)$).

The implication of this is that if we can get a better approximation for the probability of loops, $p_R(\eta_{paths}|\text{ARG}, \sigma^2, \mu)$, we may obtain better estimates.

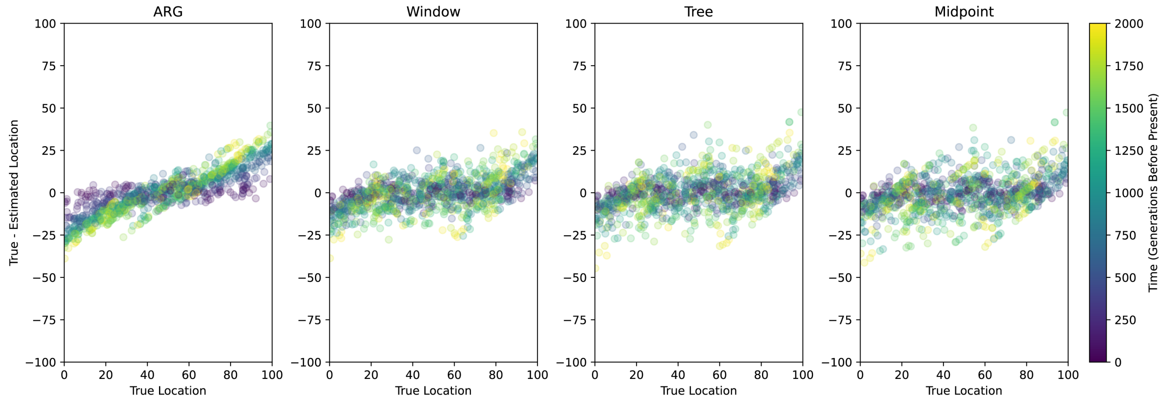

Figure S10: **Ancestor location error by time**. Error in location estimates (true - estimated locations) against the true value. The color represents the time measured backwards from present. A window of 100 trees on either side of the local tree was used for the Window panel.

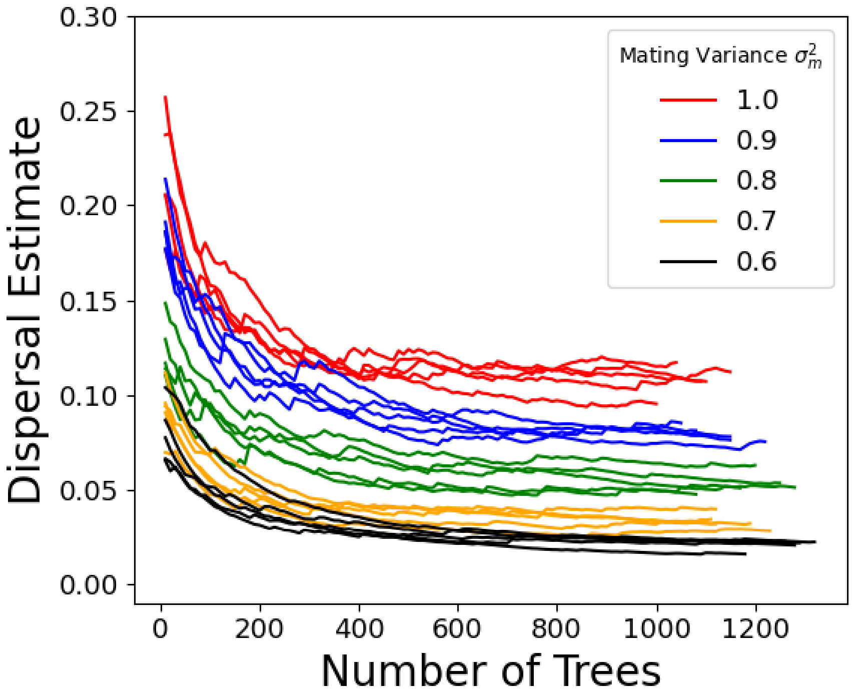

Figure S11: The maximum likelihood dispersal estimate using the unconditioned likelihood, Eq. S77.
